# Interplay between plasticity, environment and depression

**DOI:** 10.1192/j.eurpsy.2023.66

**Published:** 2023-07-19

**Authors:** I. Branchi

**Affiliations:** Center for Behavioral Sciences and Mental Health, Italian Institute of Health, Rome, Italy

## Abstract

**Abstract:**

Plasticity is the ability to modify brain and behavior, ultimately promoting an amplification of the impact of the environment on the individual’s mental health. Thus, plasticity is not beneficial per se but its value depends on contextual factors. High plasticity is beneficial in favorable, but can be detrimental in adverse living conditions, while the opposite applies to low plasticity. Consequently, resilience and vulnerability are not univocally associated to high or low plasticity. Here I will present recent findings supporting this theoretical framework and showing the role of the serotonin system in enhancing plasticity. First, we explored the Sequenced Treatment Alternatives to Relieve Depression-STAR*D dataset and analyzed the outcome of the SSRI citalopram treatment according to socioeconomic status (SES) and SSRI dosage. The results showed that SSRIs are plasticity-enhancer drugs as they amplify the influence of the living conditions on mood in a dose-dependent fashion. Second, we exploited a meta analytic approach to investigate the contribution of the serotonin-transporter-linked promoter region (5-HTTLPR) to depression vulnerability considering time as moderating factor. We found that the 5-HTTLPR x stress interaction is a dynamic process, producing different effects at different time points and confirming that individuals with high plasticity are both at higher risk and more capable to recover from depression. Overall, our findings indicate that treatments and conditions enhancing plasticity have a therapeutic value that depends on context.

**Disclosure of Interest:**

None Declared

